# A cost-effectiveness analysis for high versus standard (low) dose caffeine for the treatment of apnea in neonatal intensive care unit

**DOI:** 10.1080/20523211.2024.2345218

**Published:** 2024-05-22

**Authors:** Eilan Al-Hersh, Dina Abushanab, Fouad AbouNahia, Daniel Rainkie, Moza Al Hail, Palli Valapila Abdulrouf, Wessam El-Kassem, Daoud Al-Badriyeh

**Affiliations:** a College of Pharmacy, University of Iowa, Iowa, USA; b Department of Pharmacy, Hamad Medical Corporation, Doha, Qatar; c Neonatal Intensive Care Unit Department, Hamad Medical Corporation, Doha, Qatar; d College of Pharmacy, Dalhousie University, Halifax, Canada; e College of Pharmacy, QU Health, Qatar University, Doha, Qatar

**Keywords:** Cost-effectiveness, apnea, caffeine, intensive care unit, premature infant

## Abstract

**Objective:**

Preterm babies are prone to experiencing apnea of prematurity (AOP), mostly characterised by a pause in breathing lasting a minimum of 20 seconds. Recent literature supported higher maintenance doses of caffeine, indicating benefits. This study evaluated the cost-effectiveness of high maintenance dose (HD) versus low maintenance dose (LD) caffeine for AOP in neonates.

**Methods:**

From the hospital perspective of Hamad Medical Corporation (HMC), Qatar, a cost-effectiveness decision-analytic model was constructed to follow the use of a HD maintenance caffeine of 20 mg/kg/dose versus a LD maintenance caffeine of 10 mg/kg/dose, in a simulated cohort of AOP neonates, over a therapy follow-up duration of six weeks, until neonatal intensive care (NICU) discharge. The clinical inputs were primarily literature-based, while the resource cost and utilisation were locally extracted in HMC. The cost-effectiveness outcome measure was calculated per therapy success, defined as survival with no apnea and successful extubation removal within 72 hours, with or without adverse events. One-way and multivariate sensitivity analyses were performed to confirm the robustness of the results.

**Results:**

With 0.23 (95% CI, 0.23–0.23) enhancement in success rate, at United States dollar (US$) 3869 (95% CI, US$ 3823–3915) added infant cost, the HD caffeine was between dominant (34.8%) and cost-effective (63.7%), with an average incremental cost-effectiveness ratio of US $16,895 (95% CI, US$ 15,242–18,549) relative to LD caffeine per additional case of success. The hospitalisation contributed the most to the total infant cost, and the probability of patent ductus arteriosus was the model input that influenced the results most.

**Conclusion:**

This is the first literature economic evaluation of caffeine for AOP. Despite increasing the cost of therapy, HD maintenance caffeine seems to be a cost-effective alternative to LD caffeine in Qatar. Our results support the recent global trends of increased use of HD caffeine for AOP in NICU.

## Introduction

Preterm infants are susceptible to apnea of prematurity (AOP), a respiratory pause lasting at least 20 seconds, or less than 20 seconds if it is with bradycardia or desaturation (Eichenwald, 2016). AOP becomes more prevalent with decreasing gestational age (GA) and birth weight, affecting 100% of neonates born less than 28 weeks GA (NHSGGC Guidelines, 2023) and 85% of infants born weighing less than 1500 g (Montenegro et al., 2017). AOP was globally estimated to occur in almost all infants born at < 29 weeks GA or < 1000 g, 50% of infants born between 30 and 32 weeks, and in 7% of infants born at 34–35 weeks gestation (Mohammed et al., 2015). There is no published regional prevalence data on AOP occurrence. But, in Qatar, it is estimated that 10% of annual deliveries are < 37 weeks preterm (n = 2600). Out of which, about 15% are < 32 weeks preterm (n = 390), which were all AOP cases. Infants with AOP experience hypoxia and, potentially, hypotension (Bruschettini et al., 2023). AOP has also been associated with prolonged hospital stay and adverse neurodevelopmental outcomes (Bruschettini et al., 2023). The pathogenesis of AOP is due to the physiological immaturity of the central nervous system and decreased ventilatory drive (Bruschettini et al., 2023). The methylxanthine therapy, such as caffeine citrate, is the main pharmacological therapy used in the treatment and prevention of AOP (Aujard, 1990; Kreutzer & Bassler, 2014). Caffeine citrate acts as a central nervous system stimulant. It has been demonstrated to enhance respiratory drive, reduce the duration of ventilation and oxygen dependency, and improve disability and disability-free survival, as well as improve motor functions (Schmidt et al., 2006, 2017). According to the ‘Consensus Guidelines for Management of apnea of Prematurity UCSF Northern CA Neonatology Consortium' (Northern CA Neonatology Consortium, 2017), the standard dosing regimen of caffeine includes an intravenous (IV) loading dose of 20 mg/kg followed by an IV or oral (PO) maintenance dose of 5–10 mg/kg. However, there has been an increasing number of reports in the literature in favour of the use of higher maintenance doses of caffeine (i.e. 20 mg/kg) (Henderson-Smart & De Paoli, 2010; Vliegenthart et al., 2018), suggesting that this facilitates the removal of the endotracheal tube in mechanical ventilation (MV), reduces the duration of apnea, and decreases the AOP mortality with no significant ADRs (Steer & Henderson-Smart, 2000; Gray et al., 2011) . However, an increased dose of caffeine adds to the cost of therapy and might be associated with prolonged hospitalisation for the complete clearance of caffeine from the system, adding to a potential increase in adverse drug events (ADEs). In Hamad Medical Corporation (HMC), the primary non-profit hospital in Qatar, the use of increased doses of caffeine for AOP is in practice, but this is based on individual preference by clinicians and is not supported by any local evidence. While many studies in the literature provide important information for consideration, these do not include the economic consequences of the advantages and disadvantages of the different regimens of caffeine in AOP infants. Not only in Qatar, but internationally, there are no economic evaluations of the trade-offs between the relative clinical and economic consequences of the higher dose of caffeine. The only economic evaluation of caffeine in AOP in the literature was a study by Dukhovny et al. (2011) that compared caffeine to a placebo in AOP management.

The aim of the current study is to generate a first-time economic evidence to guide the use of the high maintenance dose of caffeine in AOP in the Qatari practice, answering the question about whether the enhanced benefits with the use of the high dose of caffeine are worth the added cost with it, relative to the lower dose of the caffeine. Consequently, our objective is to evaluate the cost effectiveness of the high maintenance dose versus the maintenance low dose of caffeine for AOP in the neonatal intensive care setting (NICU) in Qatar.

## Materials and methods

The methodology of the our study was to followed the Consolidated Health Economic Evaluation Reporting Standards (CHEERS) methods guidance (Appendix A) (Husereau et al., 2022), to perform a conventional-model-based cost-effectivness evaluation of the study dosage regimens of caffeine for AOP.

### Model structure and population

A comprehensive decision-analytic model was constructed to follow the use of high maintenance dose versus low maintenance dose of caffeine, and their potential consequences, in the management of AOP. The model included ten possible outcomes of interest. Preterm infants were differentiated based on whether they received high or low dose of caffeine. For each dose regimen, preterm infants were initially differentiated based on whether they experienced a ‘success’ or ‘failure’ health state. Success was defined as survival with less than 3 apnea episodes per day and successful extubation removal within 72 hours. Treatment success can be without adverse events (AEs) or with AEs. The AE is an undesirable or harmful outcome that develops during or after using caffeine (Voskanyan, 2018). The relevant AEs that may take place with success are intraventricular haemorrhage (IVH), periventricular leukomalacia (PVL), necrotising enterocolitis (NEC), retinopathy of prematurity (ROP), patent ductus arteriosus (PDA), bronchopulmonary dysplasia (BPD), and sepsis. Only grades 3 and 4 for each of IVH and ROP were considered, while grades 2 and 3 were considered for NEC. The remaining grades were excluded due to the assumption that these grades are mild and do not substantially affect the cost management of the infant.

**Figure 1.**
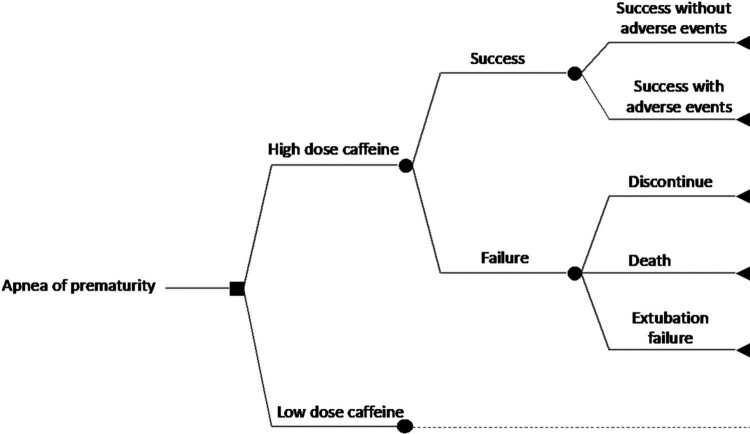
Structure diagram of the study decision tree.

In contrast, failure was defined as either (i) discontinuation of caffeine due to severe AEs, (ii) death, or (iii) extubation failure due to the persistence of apnea. Premature discontinuation is an incomplete course of caffeine due to adverse drug reactions (ADRs). The ADR of interest in the model is tachycardia, defined as a heart rate greater than 160–180 bpm (Ban, 2017). Death is defined as all-cause death during the initial NICU stay. Extubation failure is defined as the persistence of apnea after extubation removal (Kulkarni & Agarwal, 2008). The structure diagram of the study decision tree can be seen in Figure 1.

Simulating real-life uncertainty and confounding, the base case of the model was analysed based on multivariate uncertainty analysis of the model event probabilities, based on their 95% confidence interval (CI) uncertainty ranges and a triangular type of random sampling distribution.

### Study perspective and setting

As the model was developed from the public hospital perspective (i.e. HMC, the primary health care provider in Qatar). The NICU of HMC is located at Women’s Wellness and Research Center. This NICU service is a level III/IV maternal and newborn centre with a capacity of 100 beds, and it provides management and support for all extreme preterm infants, surgical, cardiac, and subspecialty cases. For the current practice for AOP management in Qatar, respiratory support and caffeine are routinely used in all newborns who are < 32 weeks GA and < 1500 grams in weight. Caffeine is also given at a higher GA if a significant apnea is developed. The caffeine is administered as a single IV loading dose, followed, after 24 hours, by a once-daily IV maintenance dose. The increased trend of using high maintenance doses of caffeine is particularly seen in newborns who are < 28 weeks and < 1000 grams. Caffeine therapy is usually continued until the newborn is off respiratory support. To note, the NICU of HMC is the only public tertiary centre (level III/IV) in Qatar. All other public and private maternal and newborn centres in the country generally follow the HMC practices for the management of AOP.

Given that the current study does not involve patients, and that the data is based on literature and available sources, no ethical approvals were required for the study undertaking.

### Comparators

The two dose regimens of caffeine that are compared in the current economic model were the low maintenance dose of caffeine, defined as 10 mg/kg, and the high maintenance dose of the caffeine, defined as 20 mg/kg. Both were intravenously administered.

### Time horizon

The model follow-up duration was until 37 weeks post-menstrual age (PMA) As per HMC practice, infants receive therapy until successful AOP mitigation by 37 weeks PMA.

### Model outcome

The trade-off between the high versus the low caffeine doses in AOP was investigated and presented via Incremental cost-effectiveness ratio (ICER) per additional case of overall success. The overall success is the rate of success with and without AEs. If dominance (i.e. lower cost and higher effectiveness) is reported; whereby, an ICER cannot be generated, the relative cost saving was reported. In the current study, the willingness-to-pay (WTP) cost-effectiveness threshold is estimated to be USD 150,000 per case of success, in line with relevant Qatari studies (Abushanab et al., 2022; Kaddoura et al., 2022; Al-Badriyeh et al., 2022).

### Measurement of model effectiveness

The main clinical input data were derivative from the meta-analysis by Chen et al. (2018). Chen et al compared the efficacy and safety of high (10–20 mg/kg daily) versus low (5–10 mg/kg daily) dose of caffeine for the management of AOP, and included 13 randomised controlled trials (RCTs) of 1515 infants younger than 37 weeks GA. Data obtained from Chen et al were the probabilities of success, death, extubation failure, PVL, and BPD. The inclusion criteria in the Chen et al study drove patient criteria in our model, mirroring the status in HMC.

Data on further consequences of events, that were not available in the Chen et al study, related to the severe tachycardia, sepsis, PDA, and ROP outcomes, were obtained from a meta-analysis by Brattstrom et al. (2019), and the IVH and NEC outcomes data was obtained from the meta-analysis by Vliegenthart et al. (2018). The Brattstrom et al study compared the efficacy and safety of high dose (loading dose >20 mg/kg and MD >10 mg/kg/day) versus low dose of caffeine (loading dose ≤20 mg/kg and MD ≤10 mg/kg/day). The study included 6 RCTs, enrolling 816 infants born before 34 weeks GA. The Vliegenthart et al study comprised 6 RCTs that included 620 infants, comparing a high versus standard dose of caffeine using an arbitrary cut-off of <10 mg/kg/day in infants with a GA <32 weeks. Like with the Chen et al study, the definition of success and the inclusion criteria in these two latter meta-analyses are consistent with those in our model, mirroring the status in HMC. Where the clinical outcomes were reported in meta-analyses as a risk ratio (RR), outcome probabilities were obtained via the equation ‘P1 = RR × P0’ (Gidwani & Russell, 2020), where RR is the risk ratio; P0 is the probability of the event in low dose caffeine-exposed infants, and P1 is the probability of the event in high dose caffeine-exposed infants.

The probability of success without AEs was not reported in the literature. Here, consistent with the locally observed trends in HMC practices, the model assumed that infants between 34 and 37 weeks GA rarely develop AEs and, hence, have AOP without AEs. In HMC, the percentage of infants who are between 34 and 37 weeks GA is reported, based on a recent local HMC study, to be 71.4% (Abushanab et al., 2022). Model outcomes, their probabilities, and their sources can be seen in Table 1.

**Table 1.** Model outcomes, their probabilities, and sources.

Parameter	High maintenance dose of caffeine	Low maintenance dose of caffeine	Source
**Success**	0.849	0.62	Chen et al. (2018)
Success without adverse events	0.0714	0.0714	Abushanab et al. (2021)
Success with adverse events	0.9286	0.9286	Abushanab et al. (2021)^a^
Success with bronchopulmonary dysplasia	0.318	0.403	Chen et al. (2018)
Success with intraventricular haemorrhage	0.065	0.057	Vliegenthart et al. (2018)
Success with patent ductus arteriosus	0.54	0.54	Brattstrom et al. (2019)
Success with necrotising enterocolitis	0.0418	0.059	Vliegenthart et al. (2018)
Success with retinopathy of prematurity	0.0421	0.0739	Brattstrom et al. (2019)
Success with sepsis	0.33	0.33	Brattstrom et al. (2019)
Success with periventricular leukomalacia	0.101	0.075	Chen et al. (2018)
**Failure**	0.151	0.38	Chen et al. (2018)^b^
Failure due to severe tachycardia	0.199	0.038	Brattstrom et al. (2019)
Failure due to death	0.076	0.104	Chen et al. (2018)
Failure due to extubation failure	0.184	0.361	Chen et al. (2018)

^a^ The probability of success with events = 1 – probability of success without event.

^b^ The probability of failure = 1 – probability of success.

### Estimating resources and costs

Given the perspective of the study, only the direct medical care costs were considered, including the costs of medications, hospitalisation, laboratory tests, diagnostic tests, and procedures (i.e. surgeries and invasive respiratory interventions), as relevant to the events.

The cost of medications was obtained from the pharmacy department of HMC. As per HMC practices, caffeine is administered as a single IV loading dose of 20 mg/kg, followed, after 24 hours, by a once-daily IV maintenance dose. The high maintenance dose regimen is 20 mg/kg/day IV, while the low standard maintenance dose regimen is 10 mg/kg/day IV. These drug maintenance dose regimens are consistent with those reported in the meta-analysis by Chen et al. (2018). However, in the Chen et al study, no exact doses were reported and, instead, maintenance doses were reported as a range of 10–20 mg/kg/dose for the high dose caffeine, and 5–10 mg/kg/dose for the low dose caffeine. For the purpose of our model, for both regimens, we used the higher end of the ranges, mirroring those used in HMC.

Clinical event costs were based on the finance department of HMC, which were available as per resource category, calculated based on a micro-costing approach of involved direct medical resources.

As per HMC practice, as discussed above, infants receive therapy until successful AOP mitigation by 37 weeks PMA. Because the median GA of preterm infants in the Chen et al meta-analysis (Chen et al., 2018) was 31 weeks, the duration of high dose and low dose caffeine administration in the model was calculated to be 6 weeks. When an infant prematurely discontinues the medication because of an ADR, the infant discontinues caffeine for 3 days before recommencing the drug administration. Based on Abushanab et al. (2022), the dose of caffeine was calculated based on an average infant weight of 1.2 Kg in HMC. Given the acute nature of the condition and the therapy, no discounting of costs was undertaken.

### Currency, price date, and conversion

The cost was reported in United States dollar (US$), based on 2023 exchange rates (1 Qatari Riyal [QAR] = 0.27 US$) and inflated to 2023 using the Consumer Price Index for Medical Care, when needed (Qatar Inflation Rate, 2023).

### Analytical methods

In addition to the uncertainty of event probabilities at the base case of the model, a one-way analysis and a multivariate analysis were performed.

- A one-way sensitivity analysis was performed by introducing uncertainty to the weight of infants at a range of – 0.2 to +0.3 Kg, using a uniform type of distribution. Also performed was a one-way sensitivity analysis that targets the acquisition cost of caffeine (−50%, + 10% uncertainty), with a triangle type of distribution. Here, a broad uncertainty range has been used as the caffeine used in HMC for AOP is a brand medication, increasing the generalisability of results to other settings and/or the future use of a generic version of caffeine.
- The multivariate sensitivity analysis was performed by introducing uncertainty to the base-case costs of events. No confidence interval was available for event costs and, therefore, an overestimated ±10% variability was used as an uncertainty range, utilising a triangular type of sampling distribution. As with the base-case analysis, the Monte Carlo simulation via @Risk-7.6® (Palisade Corporation, NY, US) was used, with 5000 iterations.

### Model validation

The model was created in Microsoft Excel. The validity of the model was examined using the Assessment of the Validation Status of Health-Economic Decision Models Checklist (Vemer et al., 2016). Additionally, a neonatologist (FA) and a health economist (DA-B) reviewed the assumptions, model structure, and findings. Independent checks were also performed by DA-B and DA in the Excel sheet to detect any modelling errors to emphasise the face validity of the modelling approach and data sources.

## Results

### Base-case clinical outcomes

The base-case analysis of the study model was based on multivariate uncertainty analysis of probability inputs. Table 2 lists the model inputs and their uncertainty distributions.

**Table 2.** Model's inputs and their uncertainty distributions.

Events	High maintenance dose of caffeine (uncertainty range, 95% CI)	Low maintenance dose of caffeine (uncertainty range, 95% CI)
**Success**	0.849 (0.764–0.913)	0.616, (0.517–0.715)
Success without adverse events	0.0714, (0.028–0.138)	0.0714, (0.028–0.138)
Success with adverse events	0.928	0.928
Success with bronchopulmonary dysplasia	0.318, (0.143–0.313)	0.403, (0.177–0.357)
Success with intraventricular haemorrhage	0.065, (0.016–0.112)	0.057, (0.011–0.099)
Success with patent ductus arteriosus	0.54, (0.284–0.482)	0.54, (0.257–0.451)
Success with necrotising enterocolitis	0.0418, (0.006–0.085)	0.059, (0.011–0.099)
Success with retinopathy of prematurity	0.0421, (0.006–0.085)	0.0739, (0.016–0.112)
Success with sepsis	0.33, (0.151–0.324)	0.33, (0.134–0.302)
Success with periventricular leukomalacia	0.101, (0.028–0.138)	0.075, (0.016–0.112)
**Failure**	0.151	0.38
Failure due to severe tachycardia	0.199, (0.331–0.532)	0.038, (0.035–0.151)
Failure due to death	0.076, (0.102–0.258)	0.104, (0.134–0.302)
Failure due to extubation failure	0.184, (0.303–0.502)	0.361, (0.621–0.805)

Note: CI: Confidence interval.

**Figure 2.**
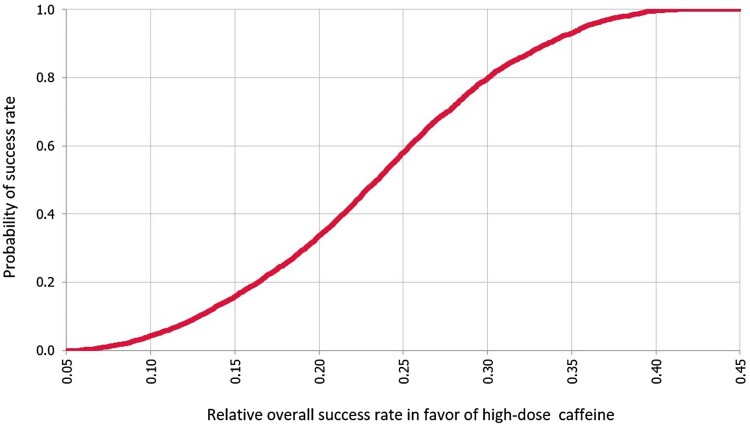
Probability curve for relative success with high dose over low dose caffeine.

In the base case, the mean probability of success with high dose caffeine is 0.849 (95% CI, 0.83–0.87), while it is 0.616 (95% CI, 0.61–0.62) with low dose caffeine. Overall, the mean effect difference in the rate of success between the high and low doses of caffeine was 0.23 (95% CI, 0.23–0.231) in favour of the high dose. The probability curve of the relative rate of success with high dose versus low dose caffeine is illustrated in Figure 2.

### Base-case economic outcomes

Table 3 summarises the cost of each study regimen, including the costs of events. Overall, the high dose caffeine has a higher cost of US$ 100,389 (95%CI, US$ 100,145–100,632), compared to low dose caffeine (US$ 96,520) (95%CI, US$ 96,322–96,717). Therefore, the mean difference in cost is US$ 3869 (95% CI, US$ 3823–3915) with the low dose over high dose caffeine.

**Table 3.** Summarised cost of each study regimen, including costs of events.

AOP treatment regimen	Outcome event	Cost (US$) of health state^a^	Proportional cost (US$) of health state	Average cost (US$) per outcome category	Total average cost (US$) of caffeine regimen
**High maintenance dose caffeine**					100,389
	**Success**			95,784	
	Success without adverse drug events	75,943	4603		
	Success with bronchopulmonary dysplasia	112,582	19,644		
	Success with intraventricular haemorrhage	117,585	4222		
	Success with patent ductus arteriosus	101,688	30,076		
	Success with necrotising enterocolitis	109,900	2522		
	Success with retinopathy of prematurity	104,131	2402		
	Success with sepsis	99,719	18,024		
	Success with periventricular leukomalacia	118,876	6592		
	**Failure**			12,279	
	Failure due to severe tachycardia	77,072	5033		
	Failure due to death	75,943	1916		
	Failure due to extubation failure	88,085	5327		
**Low maintenance dose caffeine**					96,520
	**Success**			64,341	
	Success without adverse drug events	75,853	3358		
	Success with bronchopulmonary dysplasia	112,492	16,979		
	Success with intraventricular haemorrhage	117,496	2507		
	Success with patent ductus arteriosus	101,598	20,535		
	Success with necrotising enterocolitis	109,811	2425		
	Success with retinopathy of prematurity	104,041	2879		
	Success with sepsis	99,630	12,306		
	Success with periventricular leukaemia	118,788	3335		
	**Failure**			32,179	
	Failure due to severe tachycardia	76,990	2209		
	Failure due to death	75,853	5950		
	Failure due to extubation failure	87,996	24,011		

Notes: AOP: Apnea of prematurity, US$: United States dollar.

^a^ Adjusted to the nearest 1.0.

**Figure 3.**
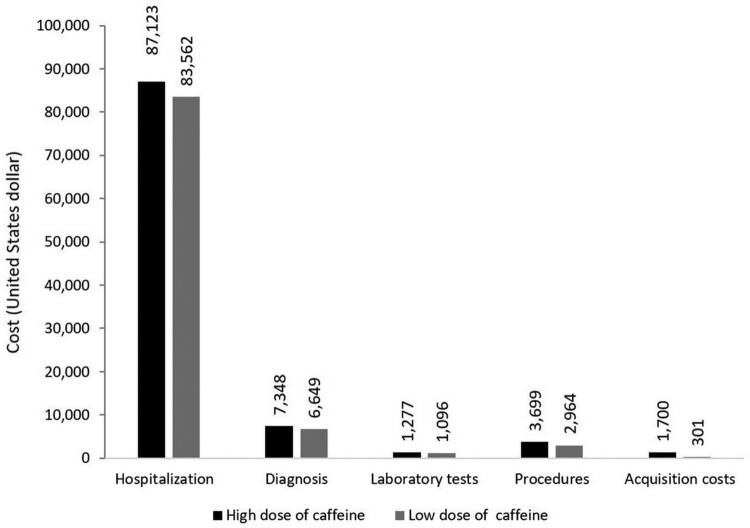
Breakdown of cost components of the caffeine regimens.

The breakdown of the cost per infant as per resource category is portrayed in Figure 3. The resource that contributes most to infant costs is hospitalisation, higher with high dose caffeine, followed by the diagnostic tests, also higher with high dose caffeine. The costs of the remaining resource categories were considerably lower for both study regimens, including the acquisition cost, which was higher with the high dose of caffeine.

### Base-case cost-effectiveness outcome

**Figure 4.**
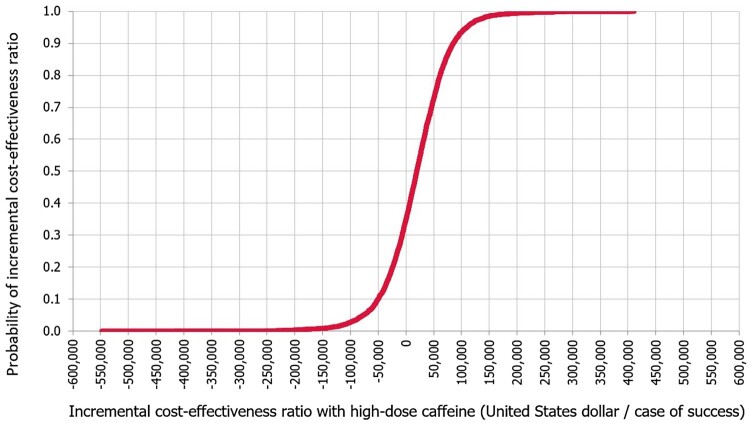
Incremental cost-effectiveness ratio P curve with high dose caffeine, base-case analysis.

The mean ICER was calculated to be US$ 16,895 (95% CI, US$ 15,242–18,549) with high dose over low dose caffeine per additional case of success. Based on the WTP threshold, the high dose of caffeine is considered cost-effective compared to low dose of caffeine. This was maintained in 63.7% of the simulated cases. In 34.8% of the cases, the high dose caffeine was dominant over the high dose caffeine. In only 1.5% of the cases, high dose caffeine was not cost effective. The ICER acceptability curve with high dose versus low dose caffeine is in Figure 4.

**Figure 5.**
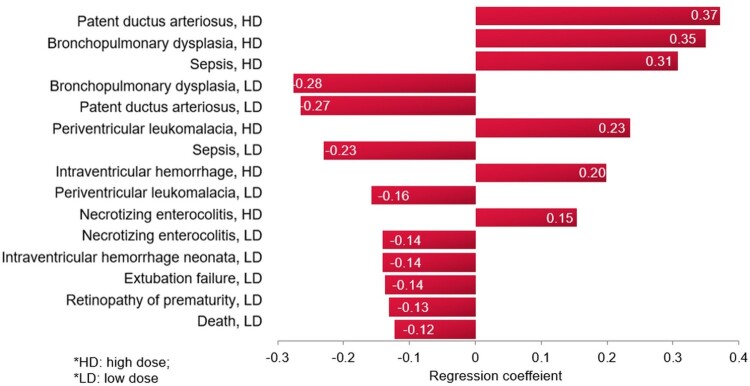
Tornado diagram of the extent of model input influence on the incremental cost-effectiveness ratio with high dose caffeine, base-case analysis.

A tornado regression analysis that ranks the models’ clinical inputs based on the strength of their association with the ICER was performed, as seen in Figure 5. The model inputs influencing the outcome the most are the probabilities of PDA, followed by BPD, and then sepsis, with the high dose caffeine.

### Sensitivity analysis

#### One-way sensitivity analysis

The one-way sensitivity analysis for the weight of infants and the caffeine acquisition cost inputs, including consequences, is presented in Table 4. The model shows robustness against the uncertainty in both inputs.

**Table 4.** Uncertainty range for variables used in one-way sensitivity analyses and main outcomes of interest.

	Analysis variables			Average high dose cost^a^, US$ (95% CI)	Average low dose cost^a^, US$ (95% CI)	ICER with high dose caffeine^a^, US$/ success (95% CI)
	Low	Base case	High			
Base case				100,389 (100,145–100,632)	96,520 (96,322–96,717)	16,895 (15,242–18,549)
Uncertainty in weight	1	1.2	1.5	106,340 (106,082–106,596)	101,734 (101,522 −101,945)	19,019 (17,415–20,622)
Uncertainty in cost of caffeine (QAR)	3.86	7.73	8.5	106,305 (106,045–106,566)	101,714 (101,502–101,926)	18,326 (16,698–19,954)

Notes: ICER: Incremental cost-effectiveness ratio, CI: Confidence interval, US$: United States dollar.

^a^ Adjusted to the nearest 1.0.

#### Multivariate probabilistic sensitivity analysis

Based on the probabilistic sensitivity analysis, the model outcome seems insensitive to potential uncertainty in the cost events as added uncertainty to the probability inputs. Following up on the costs of events presented in Table 3, the introduced event cost uncertainties and their consequences are summarised in Table 5. In addition, and in line with the base case, the high dose was cost effective over low dose caffeine in 63.5% of cases and was dominant in 34.6%. The high dose was not cost effective in only 1.9% of cases.

**Table 5.** Uncertainty ranges for variables used in multivariate sensitivity analyses and main outcomes of interest.

Model events	Event cost distribution uncertainty ±10% (US$)	Average cost of high dose caffeine per patient^a^, US$ (95% CI)	Average cost of low dose caffeine per patient^a^, US$ (95% CI)	Average ICER with high dose caffeine^a^, US$ (95% CI)
Success without adverse drug events	(68,339–83,525)	106,315 (106,050–106,579)	101,716 (101,501–101,930)	18,801 (18,280–19,321)
Success with bronchopulmonary dysplasia	(101,309–123,823)			
Success with intraventricular haemorrhage	(105,812–129,325)			
Success with patent ductus arteriosus	(91,506–111,841)			
Success with necrotising enterocolitis	(98,896–120,873)			
Success with retinopathy of prematurity	(93,705–114,528)			
Success with sepsis	(89,734–109,676)			
Success with periventricular leukomalacia	(106,974–130,745)			
Failure due to severe tachycardia	(69,356–84,768)			
Failure due to death	(68,339–83,525)			
Failure due to extubation failure	(79,266–96,881)			

Notes: ICER: Incremental cost effectiveness ratio, CI: Confidence interval, US$: United States dollar.

^a^ Adjusted to the nearest 1.0.

**Figure 6.**
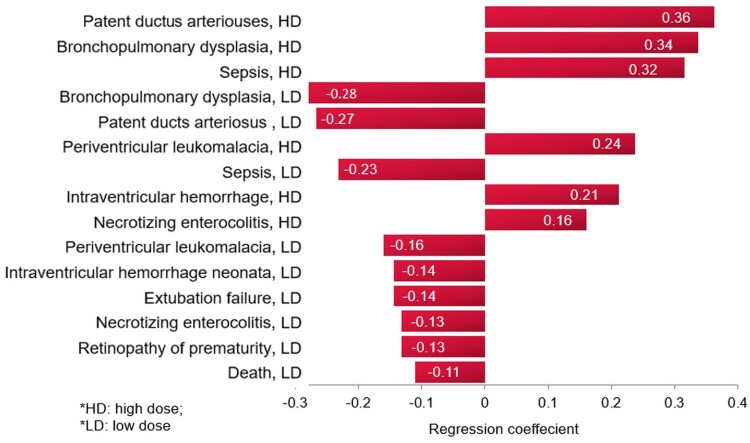
Tornado diagram of the extent of model input influence on the incremental cost-effectiveness ratio with high dose caffeine, multivariate sensitivity analysis.

A tornado regression analysis of the model inputs, including costs of events, based on the probabilistic sensitivity analysis (Figure 6), demonstrated that the rank of the model inputs as per their influence on the ICER of the model did not change from the base-case scenario, with the top influencing probabilities being those for PDA and BPD with the caffeine.

### Model validation

The AdViSHE checklist is presented in Appendix 2.

## Discussion

Internationally, the optimal regimen of caffeine for the treatment of AOP in NICUs is highly controversial and remains unclear as there are no universal guidelines or consensus regarding the most appropriate dosing approach. The uncertainty surrounding the relative variability in the effectiveness and safety performance of different regimens is further amplified when also considering the relative economic impact. In the medical literature, to date, there has yet to be a clear, comprehensive evidence that includes the economic impact for guiding the comparative regimens for AOP, including in Qatar. Our study is the first economic evaluation that compared high dose versus low dose of caffeine for the treatment of AOP in NICU.

The multivariate base-case results from the comparative simulation-based cost-effectiveness model in this current research illustrated a difference in the probability of success (0.23 [95% CI, 0.23–0.231]) in favour of high dose compared to low dose caffeine. For the cost difference, this was less than US$ 4000 in favour of the low dose caffeine. While the proportional cost associated with the success outcomes was over US$ 30,000 higher with high dose caffeine, this is considerably minimised by around US$ 20,000 proportional cost in favour of the high dose caffeine associated with the failure (Table 3). Taking the cost difference into consideration, the high dose caffeine was between cost effective and dominant in 98.5% of cases.

There are no other comparative economic values of the high and low doses of caffeine in the literature to contrast our current results to. However, dosage strategy wise, and due to the fact that there is no international consensus to guide the use of the high dose caffeine in AOP, different preferences are reported in different settings, including regionally. In one study in Saudi Arabia (KSA), Ansari et al. conducted a survey among physicians to collect data on the use of caffeine in the management of AOP across hospitals in KSA. The survey revealed that the widely preferred dosing regimen is a loading dose of 20 mg/kg followed by a maintenance dose of 5 mg/kg/day, which maintains therapeutic plasma caffeine concentrations. The maintenance dose can be adjusted based on changes in body weight, and if apnea persists despite treatment, the daily maintenance dose can be increased to a maximum of 10 mg/kg/day. This is all in the range of the standard low dose (Al Ansari et al., 2018). This is in contrast to that reported in another study in Egypt, by Mohammad et al., which, like ours, proposed a high maintenance caffeine dose of 20 mg/kg/day as the better dose, associated with a significant reduction in extubation failure, frequency of apnea, and days of documented apnea. In this study, however, the loading dose was up to 40 mg/kg (Mohammed et al., 2015). Internationally, while the Consensus Guidelines for Management of Apnea of Prematurity by the Northern CA Neonatology Consortium, as an example, recommend a loading dose of 20 mg/kg intravenously and a maximum maintenance dose of 10 mg/kg intravenously or orally (Alhersh et al., 2020), both the Starship Children's Hospital and The Royal Children's Hospital Melbourne guidelines, as other examples, recommend a high maintenance dose of 20 mg/kg of caffeine, administered intravenously or orally, if apnea persists (Starship Children's Hospital, 2023; The Royal Children's Hospital Melbourne, 2024). In any case, regardless of variations in practices, there is a trend of increased use of higher doses of caffeine for AOP. In a recent systematic review of meta-analyses that was conducted by our group, the higher doses of caffeine (>20 mg/kg/day) were generally associated with greater effectiveness against various outcomes, but the quality of the systematic reviews and meta-analyses varied (Alhersh et al., 2020).

In our study, the one-way and multivariate uncertainty analyses confirmed the high dose caffeine as the favourable option in comparison to low dose caffeine over a range of variabilities in cost inputs and event probabilities. Further establishing the importance of looking at secondary costs of therapies, in addition to their acquisition costs, our breakdown analysis indicated that over 80% of the cost per infant with either regimen is hospitalisation costs, followed by diagnostic costs.

In the multivariate base case, according to the results from the tornado regression analysis, the most influential model inputs on the study outcome were the probability of BPD and PDA. This is not unanticipated because, while both BPD and PDA are not considerably different from other events in terms of costs, they have the highest probabilities in the model. In contrast, the success with no AEs has a minimal impact on outcome, which is because while it is associated with a relatively low probability of occurrence, it is also associated with the lowest cost among events, about 67% of the cost of BPD. In any case, all sensitivity-analysis variations in the model inputs, including the most influential, did not change the overall conclusion of the study.

A recent Cochrane review of 7 trials (894 preterm infants born <37 weeks’ gestation) compared the effect of high dose strategy versus standard-dose (i.e. low) strategy of caffeine, for the prevention and treatment of AOP peri-extubation, on the all-cause death prior to hospital discharge and the major neurodevelopmental disability in the infants (Bruschettini et al., 2023). Consistent with our model, high dose strategies were defined as a high loading dose (i.e. > 20 mg/kg) or a high maintenance dose (i.e. > 10 mg/kg/day), and standard-dose strategies were defined as a standard loading dose (i.e. ≤ 20 mg/kg) or a standard MD (i.e. ≤ 10 mg/kg/day). While the review concluded that high dose strategies may have little or no effect on reducing all-cause death or long-term neurodevelopmental disability, the review did not evaluate the impact of different doses of caffeine on immediate outcomes, including reduction in apnea episodes and successful extubation removal, as well as AEs. The model in our study was populated with data to a different extent from different sources (i.e. meta-analyses) available in the literature (Vliegenthart et al., 2018; Chen et al., 2018; Brattstrom et al., 2019), which was to account for missing data in each of the individual sources. The inclusion criteria of neonates are very similar to the patient population included for caffeine treatment of apnea in the local HMC setting. It is important to note that the primary outcome reported, i.e. the success of apnea treatment, is the same outcome used by decision-makers to follow-up on infants at HMC, which is defined as infants who continued living with the less than 3 apnoeic spells per day with a successful tracheal tube removal (Chen et al., 2018). Also important is that the regimens of study medications in the meta-analyses mirror those used in the HMC for the treatment of AOP. The main clinical input values were extracted from a comprehensive meta-analysis, by Chen et al. (2018). Chen et al study is a strength as it is a most comprehensive meta-analysis, involving 13 RCTs of head-to-head comparisons between high dose and low dose caffeine. In addition, it included infants with apnea who are less than 37 weeks GA, which is identical to the targeted population in the current study.

Another strength of this study is how comprehensive the decision-analytic model is. The model represents all the possible consequences of using caffeine to treat AOP, including AEs that do not constitute failure and, hence, an overall cost of resource utilisation that is more accurately represented. Added to the strengths is that the current comparative model could simulate a follow up of infants until discharge as per HMC practices, i.e. 6 weeks of follow up until 37 weeks PMA.

The therapy outcome of interest, as per local practices, is the absence of apnea with/without AEs (i.e. BPD, sepsis, NEC, IVH, PVL, and ROP) or failure due to (severe tachycardia, extubation failure, and death). Only grades 3 and 4 IVH and ROP and grades 2 and 3 NEC were of interest as these outcomes are particularly expected to significantly affect infants’ management cost, with the lower grades not usually requiring management.

The current cost-effectiveness project is a hospital quality assessment type of research, and its benefits are more relevant to the best NICU practices and specific recommendations, to do with caffeine use in AOP, rather than being related to the broader scope of policies and governing procedures. The primary clinical significance of our study findings is that they confirm the appropriateness of the recent trends, in Qatar and internationally, of increased use of high maintenance doses of caffeine in AOP. In Qatar, the patient implication of this is particularly important for infants with extremely low to very low birth weights, where increased doses of caffeine would benefit the most, improving outcomes, reducing AEs, particularly the BPD and PDA, and reducing hospital stay. As indicated earlier, there is an increased interest in using high maintenance doses of caffeine in newborns who are < 28 weeks GA and weigh < 1000 grams in Qatar. Now, based on our results, the proposed is to have all newborns who are < 28 weeks GA and weigh < 1000 grams universally start the high caffeine dose therapy. If this proves beneficial, disagreements between practitioners will cease to exist, for the high dose caffeine to become the new universal routine practice for AOP, including those with > 28 weeks GA and weight > 1000 grams in HMC. This brings to light another aspect of practice for enhancement, to do with the fact that the current practice in Qatar does not recommend the therapeutic drug monitoring (TDM) when administering caffeine in the NICU of HMC. While this is consistent with the recommendation against routine TDM of standard caffeine treatment by the American Academy of Pediatrics Committee on Fetus and Newborn (Eichenwald, 2016), as example, future directions in the Qatari practice should consider the serum monitoring of caffeine when administration of high doses increases. This is supported by the National Institute for Health and Care Excellence (NICE) guidelines in the UK, as another example, suggesting measuring plasma levels when high dose caffeine is utilised (Specialist neonatal respiratory care for babies born preterm NICE guideline, 2019). This is important, especially when thinking about severe tachycardia, as one of the barriers that limits the clinical application of high doses, whereby severe tachycardia is associated with high-dose caffeine. Within this context, high dose should be monitored and used with caution.

There are several limitations that need to be acknowledged in the current study. One limitation is that the probabilities of the clinical events in the decision-analytic model were obtained from meta-analyses. While this comes with the advantage of relying on a well-established methodology that allows more accurate estimation of an effect compared to single studies, due to the increased amount of data and statistical power (Stone & Rosopa, 2017), the use of meta-analyses comes with important limitations to the economic evaluation. First, there is the jeopardised generalisability of results to the local setting due to differences in neonate demographic characteristics; none of the meta-analysis studies included Qatari-based research as an example. Second, the specific treatment durations that are pre-defined by the meta-analysis might limit knowledge of important consequences and outcomes that could influence the overall cost of therapy. Nonetheless, it is important to emphasise that, as indicated in the methods, important elements such as the patient criteria, the definition of success, caffeine doses, and the duration of patient follow-up in the meta-analyses mirrored the status in HMC, which enhances confidence in the meta-analysis sources used. The inherent uncertainties and variable confounding associated with the extracted clinical inputs, however, still exist and cannot be underestimated. In response to such uncertainty, the base-case of our model was based on multivariate uncertainty analysis of input data (Abushanab et al., 2022; Al-Shaibi et al., 2023; Al-Badriyeh et al., 2022), with uncertainties in various probabilistic values randomly interacting, just like in the real-life situation. Furthermore, when additional uncertainty was added as part of one-way and multivariate sensitivity analyses, the study conclusion was insensitive to the uncertainty in all key input variables.

Also, a limitation, and despite this study’s robustness against uncertainty, the results of this study should not be easily extrapolated to infants in different settings, especially due to variations in the resource utilisation and nature of the healthcare system In our study, the head-to-head clinical and resource utilisation data were not locally based. Indeed, outcomes in the current study can be best confirmed through a follow-up future prospective or retrospective data collection that evaluates the comparative clinical and economic impacts of caffeine for the neonatal apnea. This is important as it will validate the findings of our simulation model using real-world data from clinical settings in Qatar to assess actual success rates and other outcomes associated with high-dose and low-dose caffeine citrate, including resource use. However, this is currently difficult, mostly due to the relatively low number of neonates who received high dose caffeine. Therefore, locally specific simulation studies, like the current ones, are considered important for the decision guidance in local practices. Here, high dose caffeine has been demonstrated to be mostly superior to low dose caffeine in terms of the trade-off between clinical and economic consequences of the caffeine.

Further to limitations, there is no approved WTP cost-effectiveness threshold in Qatar. While the World Health Organization (WHO) suggests using 1–3 times the GDP per capita as the value of the threshold in a country, it is acknowledged that this is arbitrary and not based on any methodological justification (Cameron et al., 2018). In addition, the average 2022 GDP per capita in Qatar was approximately US$114,648 (The World Bank, 2022); one of the world’s highest. Thus, adopting the WHO recommendation for calculating the WTP will result in a range of values that is too wide to be directly useful, i.e. US$ 114,648–343,944. In this study, and in line with previous Qatar-based publications (Abushanab et al., 2022; Al-Badriyeh et al., 2022; Kaddoura et al., 2022), we adopt a threshold value of US$ 150,000, which is increasingly accepted as a higher threshold value in the literature, which is also within the range suggested by the WHO for Qatar (Marseille et al., 2015).

Lastly, our model focuses on short-term outcomes only, whereby it is also important to investigate the long-term effects of high-dose versus low-dose caffeine citrate in preterm infants with AOP. Future research could assess the impact of these treatment strategies on long-term neurodevelopmental outcomes. Long-term follow-up studies would provide a more comprehensive evaluation of the cost-effectiveness and clinical effectiveness of the interventions.

Another potential area for future investigation could be exploring potential heterogeneity in treatment effects and cost-effectiveness across different subgroups of preterm infants. Factors such as gestational age, birth weight, severity of apnea, and comorbidities may influence the effectiveness and cost-effectiveness of high-dose versus low-dose caffeine citrate. Conducting subgroup analyses can provide insights into the differential impacts of treatment strategies and help identify the populations that may benefit the most from each approach.

Furthermore, preliminary studies on genetic factors have shown an association between genetic polymorphisms and the clinical response to caffeine therapy (Guo et al., 2022). Further research is needed to understand the role of genetic variants in the response to caffeine therapy in premature infants.

## Conclusion

In conclusion, this is the first economic evaluation of caffeine for AOP in the literature, demonstrating that despite an anticipated increase in the therapy cost with the high maintenance dose caffeine, this was between cost-effective and dominant over low maintenance dose caffeine in 98.5% of cases. Therefore, considering the assumptions and limitations made in our research, the results support the use of high dose over low dose caffeine for the treatment of AOP in the state of Qatar.

## Supplementary Material

Supplemental Material
