# Supplementary material for: A cost-effectiveness analysis for high versus standard (low) dose caffeine for the treatment of apnea in neonatal intensive care unit
Source: J Pharm Policy Pract. 2024 May 22;17(1):2345218. doi: 10.1080/20523211.2024.2345218 (PMC11123466; doi:10.1080/20523211.2024.2345218)
Supplement: Supplemental Material [file JPPP_A_2345218_SM4066.zip › suppl_data/Appendix 2.docx]

**Appendix 2.** Assessment of the Validation Status of Health-Economic Decision Models

| Question |  | |
| --- | --- | --- |
| Part A: Validation of the conceptual model | |  |
| A1/ Face validity testing (conceptual model): Have experts been asked to judge the appropriateness of the conceptual model? | | ✓ (DA-B) |
| A2/ Cross validity testing (conceptual model): Has this model been compared to other conceptual models found in the literature or clinical textbooks? | | N/A |
| Part B: Input data validation | |  |
| B1/ Face validity testing (input data): Have experts been asked to judge the appropriateness of the input data? | | ✓ (DA-B) |
| B2/ Model fit testing: When input parameters are based on regression models, have statistical tests been performed? | | N/A |
| Part C: Validation of the computerized model | |  |
| C1/ External review: Has the computerized model been examined by modelling experts? | | N/A |
| C2/ Extreme value testing: Has the model been run for specific, extreme sets of parameter values in order to detect any coding errors? | | ✓ |
| C3/ Testing of traces: Have patients been tracked through the model to determine whether its logic is correct? | | ✓ |
| C4/ Unit testing: Have individual sub-modules of the computerized model been tested? | | ✓ |
| Part D: Operational validation | |  |
| D1/ Face validity testing (model outcomes): Have experts been asked to judge the appropriateness of the model outcomes? | | ✓ (DA-B and FA) |
| D2/ Cross validation testing (model outcomes): Have the model outcomes been compared to the outcomes of other models that address similar problems? | | ✓ |
| D3/ Validation against outcomes using alternative input data: Have the mod | | ✓ |
| D4/ Validation against empirical data: Have the model outcomes been compared to empirical data? | | ✓ |
| Part E: Other validation techniques | |  |
| E1/ Other validation techniques: Have any other validation techniques been performed? | | N/A |
